# Evaluation of alpha-gliadin celiac disease epitopes in some *Aegilops* species containing the D genome

**DOI:** 10.1186/s12870-026-08966-0

**Published:** 2026-05-20

**Authors:** Rahman Ebrahimzadegan, Ghader Mirzaghaderi

**Affiliations:** Department of Plant Production and Genetics, Faculty of Agriculture, University of Kurdistan, Sanandaj, Iran

**Keywords:** *Aegilops*, D-genome, Celiac disease, Amplicon sequencing, Alpha-gliadin, Immunogenic peptides

## Abstract

Wheat gluten protein contains toxic peptides that trigger celiac disease in genetically susceptible individuals. These toxic peptides are primarily found in gliadin fractions, which are largely derived from the D subgenome of allohexaploid wheat. *Aegilops* species are valuable germplasm resources for wheat breeding. In this study, we analyzed the content and composition of alpha-gliadin CD epitopes in ten genotypes from five distinct species of the *Aegilops* genus: *Aegilops tauschii*, *Aegilops crassa*, *Aegilops juvenalis*, *Aegilops cylindrica*, and *Aegilops ventricosa*. This analysis was conducted using RNA sequencing of the main amplicon of alpha-gliadins that includes the most toxic CD peptides. Our results showed significant differences in the types and distribution patterns of identified CD epitopes across various species and accessions. All the canonical and the most toxic CD epitopes were detected in the studied *Aegilops* genotypes. However, the intact form of the 33-mer region was absent in all genotypes. *Ae. ventricosa* ‘AE 1511’ and *Ae. juvenalis* ‘AE 537’ exhibited the highest expression frequency for the p31-43 and DQ2.5-glia-α3 epitopes, categorizing them as the most toxic species. In contrast, *Ae. crassa* ‘TA1873’, *Ae. cylindrica* ‘AE 1658’, and *Ae. crassa* ‘AE 815’ showed the highest expression frequencies for DQ2.5-glia-α1a and DQ2.5-glia-α2, identifying them as the least toxic species. Our findings suggest that incorporating the D genome from *Ae. crassa* and *Ae. cylindrica* ‘AE 1658’ into wheat breeding programs might be a promising strategy for reducing gluten toxicity associated with celiac disease.

**Supplementary Information:**

The online version contains supplementary material available at 10.1186/s12870-026-08966-0.

## Key message

The D genome of *Aegilops crassa* and *Aegilops cylindrica* ‘AE 1658’ contains a relatively lower amount of toxic celiac disease epitopes than that of other *Aegilops* species.

**Supplementary Information:**

The online version contains supplementary material available at 10.1186/s12870-026-08966-0.

## Introduction

Bread wheat (*Triticum aestivum*), an allohexaploid species (2*n* = 6*x* = 42; AABBDD), is a significant agronomic cereal worldwide. It is valued for its protein content (8–15%) and plays an essential role in the diets of large populations. The main component of wheat proteins is gluten, which is a vital constituent for the production of various end products [1]. Gluten consists of polymeric glutenins and monomeric gliadins. Glutenins can be separated by SDS-PAGE into high molecular weight and low molecular weight glutenin subunits (HMW-GSs and LMW-GSs), while gliadins are more effectively resolved on Acid-PAGE, comprising four distinct groups: α/β-gliadins, γ-gliadins, and ω-gliadins [2].

Although both gluten fractions are vital for the breadmaking quality of wheat flour, certain epitopes found specifically in gliadin subunits resist the digestion process in the human gastrointestinal system and trigger immunogenic responses in individuals susceptible to celiac disease (CD), leading to significant gastrointestinal issues [3, 4]. Celiac disease affects 0.7–2.9% of the global population, with increased prevalence in females, autoimmune patients, and relatives of affected individuals [5]. Currently, the only solution to prevent symptoms of celiac disease in susceptible patients is to follow a strict gluten-free diet, which is challenging due to gluten’s viscoelastic properties essential in the processing of many food products [6, 7].

The immunogenic epitopes/peptides contain a conserved core consisting of nine amino acids, which are presented to CD4 T cells by the human leukocyte antigen haplotypes HLA-DQ2 and HLA-DQ8. This process triggers an immune response within the small intestine of affected individuals, leading to a variety of gastrointestinal complications, including intestinal inflammation, villus atrophy, and nutrient malabsorption. Among the various HLA-DQ molecules, the HLA-DQ2.5 haplotype is associated with an increased risk due to its ability to form more stable complexes with T-cell epitopes. To date, numerous epitopes associated with HLA-DQ2.5 have been discovered within gluten proteins, primarily located in the gliadin fraction [4, 8].

**Fig. 1:**
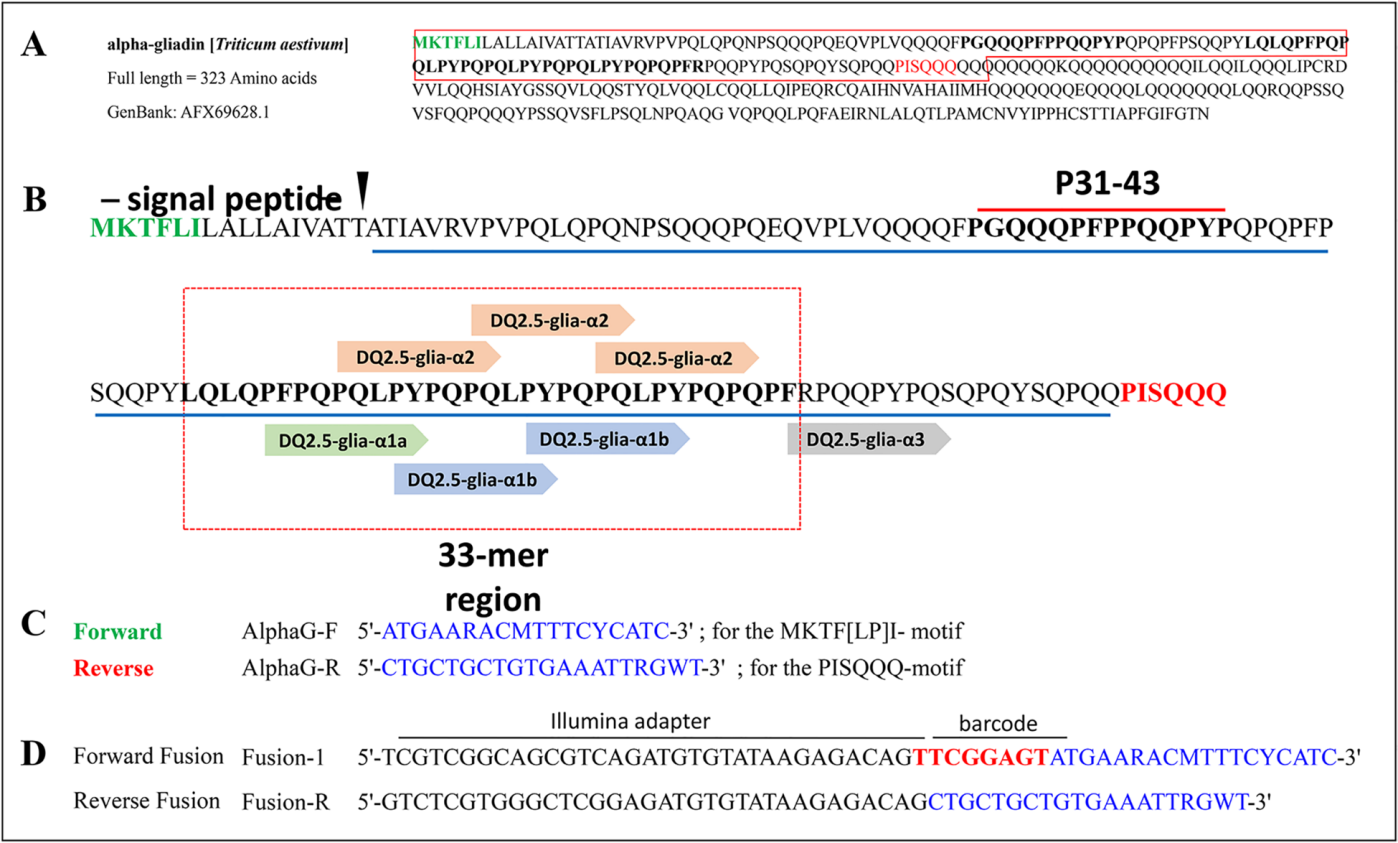
Amino acid sequence, first repetitive toxic domain and primers characteristics of a typical alpha-gliadin from *Triticum aestivum* (AFX69628.1). **A** The full-length alpha-gliadin sequence. **B** The first variable alpha-gliadin domain possessing canonical and toxic CD epitopes including P31-43, DQ2.5-glia-α1, -α2 and -α3. MKTFLI (in green) and PISQQQ (in red) motifs show the position of the forward and reverse PCR primers (**C**) used for the amplification of this variable domain, respectively. The 33-mer sequence represents the most toxic region of alpha-gliadin containing canonical CD epitopes indicated in dashed rectangle. **C** Sequences of forward and reverse primers designed for the alpha-gliadin MKTFLI (in green) and PISQQQ (in red) motifs. **D** The Fusion forward primers are specific to each genotype (one example is shown here), and include a sequence for Illumina sequencing (Illumina adapter), a genotype-specific identifier barcode (Table 1, for multiplexing and identifying of different read corresponding to each genotype), and alpha-gliadin forward primer. Reverse primer is universal for all genotypes and contains only the Illumina adapter and the alpha-gliadin reverse primer

Within gluten fractions, toxic epitopes are primarily found in α-, γ-, and ω-gliadins, and with lesser amounts in LMW-GSs. Among these, α-gliadins are the major components responsible for celiac disease. Alpha-gliadins belong to a multigene family that consist of 25 to 150 members per haploid genome of hexaploid wheat, located at the *Gli-2* loci on the short arms of the homoeologous group 6 chromosomes. Most of these genes are believed to be pseudogenes due to the presence of internal stop codons in their polynucleotide sequences [9]. Alpha-gliadins contain three key and canonical immunogenic epitopes relevant to celiac disease: the p31-43 peptide, which triggers a non-T cell-dependent immune response; the 33-mer peptide, which is highly immunogenic with six copies of two toxic HLA-DQ2.5 epitopes (DQ2.5-Glia-α1 and DQ2.5-Glia-α2); and the less immunogenic DQ2.5-Glia-α3 peptide, which overlaps with the 33-mer region (Fig. 1A,B). Among the canonical epitopes, DQ2.5-Glia-α1 is the primary inflammatory trigger in most CD patients. These toxic peptides are genome-specific and are primarily located at the Gli-2 locus of the D-subgenome in hexaploid wheat, which is believed to have originated from *Ae. tauschii* during its evolution [10–13].

Recent origination of allohexaploid wheat from limited natural hybridization between *T. turgidum* (AB-genome) and *Aegilops tauschii* (D-genome) [14], coupled with selection for increased yield performance, has affected the genetic diversity of common wheat especially in its D genome [15]. Moreover, incorporating the D subgenome from various *Aegilops* species into wheat can modify the genomic content and gene expression profiles associated with gluten proteins relevant to celiac disease. Since the content and expression levels of toxic CD epitopes in gluten are directly linked to increased allergenicity [16], precise measurement of these unwanted epitopes is vital for evaluating *Aegilops* species with relatively safe gluten. Currently, the partial sequencing of alpha-gliadin RNA amplicons/regions, which are the major sources of toxic CD epitopes, has shown great potential for assessing the content and expression levels of celiac disease epitopes across different wheat and *Aegilops* genotypes [11, 17, 18].

Here, we analyzed the content and composition of alpha-gliadin CD epitopes across ten genotypes from five *Aegilops* species including *Ae. tauschii*, *Ae. crassa*, *Ae. juvenalis*, *Ae. cylindrica*, and *Ae. ventricosa*. We utilized RNA amplicon sequencing of the major toxic region of alpha gliadins to achieve our objectives. Identifying *Aegilops* species with lower amounts of toxic CD epitopes may provide a valuable resource for developing hexaploid wheat cultivars that are tolerable for individuals susceptible to celiac disease.

## Materials and methods

### Plant materials

Ten *Aegilops* genotypes, all possessing the D-genome, were obtained from the IPK Gene Bank (Germany). These genotypes represent five species (with two accessions each): *Aegilops tauschii* (*Ae. tauschii* ‘AE 1600’ and *Ae. tauschii* ‘AE 1211’)*, Aegilops crassa* (*Ae. crassa* ‘TA1873’ and *Ae. crassa* ‘AE 815’)*, Aegilops juvenalis* (*Ae. juvenalis* ‘AE 537’ and *Ae. juvenalis* ‘AE 1495’)*, Aegilops cylindrica* (*Ae. cylindrica* ‘AE 1658’ and *Ae. cylindrica* ‘AE 1658’)*, and Aegilops ventricosa* (*Ae. ventricosa* ‘AE 1511’ and *Ae. ventricosa* ‘AE 730’) (Table 1). For each genotype, 20 seeds were placed in a Petri dish lined with Whatman filter paper, moistened with 5 mL of tap water, and incubated at room temperature in the dark to ensure uniform germination. After 2 to 3 days, germinated seeds were transplanted into 10 × 10 cm pots filled with garden soil and transferred to a growth chamber with a photoperiod of 16 h of light and 8 h of dark. Seedlings were maintained under these conditions for two weeks until they reached the two-leaf stage. Following this period, the seedlings were subjected to vernalization at 4 °C for two months. For each genotype, four seedlings were cultured in three pots (20 × 21 cm) filled with a 1:1:1 mixture of clay, sand, and organic fertilizer. These pots were kept in a greenhouse under the same photoperiod of 16-h light and 8-h dark, with night and day temperatures set at 16 °C and 22 °C, respectively. All pots were watered twice weekly and maintained under these conditions until the initiation of flowering and subsequent seed sampling.

**Table 1:** Genotypes, genome composition, genotype identifier barcodes, and Gene bank location of *Aegilops* genotypes used in this study

Code	Genotypes	Genome	Genotype-specific identifier barcode	Gene bank
1	*Ae. juvenalis* ‘AE 537’	DDMMUU	TTCGGAGT	IPK^*^
2	*Ae. juvenalis* ‘AE 1495’	DDMMUU	ACTCATTT	IPK
3	*Ae. tauschii* ‘AE 1600’	DD	GGGATCCG	IPK
4	*Ae. tauschii* ‘AE 1211’	DDDD	CAAGATAA	IPK
5	*Ae. cylindrica* ‘AE 1594’	CCDD	GGACAACG	IPK
6	*Ae. cylindrica* ‘AE 1658’	CCDD	AGCGAGCT	IPK
7	*Ae. ventricosa* ‘AE 1511’	D^v^D^v^N^v^N^v^	CTGCACGT	IPK
8	*Ae. ventricosa* ‘AE 730’	D^v^D^v^N^v^N^v^	GCACTAGT	IPK
9	*Ae. crassa* ‘TA1873’	D^1^D^1^X^cr^X^cr^	TACTGAGA	WGRC^**^
10	*Ae. crassa* ‘AE 815’	D^1^D^1^X^cr^X^cr^	ATCGTACT	IPK

^*^Leibniz Institute of Plant Genetics and Crop Plant Research (IPK), Gatersleben, Germany

^**^Wheat Genetic Resources Centre, Manhattan, Kansas State University, United States

### RNA extraction and cDNA preparation

Immature seeds were collected at milk to soft dough stage (approximately 21 days after flowering), and immediately frozen in liquid nitrogen within 2 mL microtube. The samples were then stored at $-$ 80 °C for subsequent RNA extraction and cDNA preparation. RNA was extracted using the SDS-LiCl method with a slight modification [19]. Briefly, RNA was extracted from at least five seeds (100 mg) of each *Aegilops* plant. This was done by adding 600 µl of the extraction buffer containing 2% SDS, followed by phase separation through the addition of an equal volume of phenol: chloroform: isoamyl alcohol mixture (25:24:1) to ensure isolation of RNA from protein and DNA. Isolated RNA was then precipitated by adding 20 µl of 8 M LiCl and incubating the mixture at $-$ 20 °C for at least 2 h. The RNA was then washed with 75% ethanol and air-dried under a laminar flow hood for 5 min. Finally, the RNA was dissolved in 20 µl of DEPC-treated water. To assess the quality and quantity of the extracted RNA, 2 µl of RNA from each sample was analyzed using 1.2% agarose gel electrophoresis (Supplementary Fig. 1 A). To prevent amplification from genomic DNA during PCR, RNA samples were treated with DNase I. For each genotype, three biological replicates of RNA samples were used for cDNA preparation. After RNA quantification with a Nanodrop spectrophotometer, 500 ng of RNA from each sample was used to synthesize cDNA using the TaKaRa kit (Otsu, Shiga, Japan), following the manufacturer’s instructions.

### Amplification of alpha-gliadins transcripts and amplicons sequencing

For each genotype, alpha-gliadins transcripts/amplicons (Fig. 1A, B) were amplified from three biological replicates of cDNA in two rounds of PCR reactions. The first PCR reaction was performed using the previously reported of the forward and reverse alpha-gliadin specific primers [17] including AlphaF (5′-atgaaracmtttcycatc-3′; for the MKTF[LP]I- motif) and AlphaR (5′- ctgctgctgtgaaattrgwt-3′; for the PISQQQ-motif), respectively (Fig. 1C; Supplementary Fig. 1B). These primers amplify the repetitive domain of a typical alpha gliadin gene encoding the most immunogenic CD epitopes in wheat gluten protein including the p31-p43 epitope, 33-mer fragment associated epitopes (Fig. 1B, see dashed rectangle), and DQ2.5-Glia-α3 (Fig. 1B, underlined in blue). To higher the amplification accuracy, PCR reaction was repeated in two different thermocyclers. The first PCR reaction was prepared in a total volume of 10 µl, containing 25 ng of template cDNA, 5 pmol of each primer, 2.5 mM of each dNTP, 2.5 mM of MgCl2, and 0.5 U of Taq polymerase. The cycling parameters for the PCR included an initial denaturation step at 94 °C for 5 min, followed by 30 cycles of 30 s at 94 °C, 15 s at 50 °C, and 30 s at 72 °C, concluding with a final extension at 72 °C for 5 min (Supplementary Fig. 1B). After the initial PCR, the PCR product for each genotype was diluted 1:10 in nuclease-free water. A second round of PCR was then performed in 10 µl reactions using fusion primers (Fig. 1D; Supplementary Fig. 1 C), with 1 µl of this diluted product serving as the template DNA. The fusion primers included sequences necessary for Illumina sequencing. The forward fusion primer also contained an 8 bp identification sequence (barcode) unique to each genotype to facilitate multiplexing and subsequent separation of sequencing raw reads, along with a gene-specific region. In contrast, the reverse fusion primer was universal for all genotypes and did not contain a specific barcode (Fig. 1D). The conditions for the second PCR reaction were similar to those used in the first PCR. Following the second PCR reaction, PCR products from all biological replicates per plant were pooled (each 5 µl). Because the second PCR incorporated genotype-specific barcode sequences into the amplicons, the alpha-gliadin amplicons were sequenced using a multiplex sequencing approach. For this purpose, the pooled PCR products from all genotypes were combined (10 µl per genotype), and a total of 100 µl of the mixture was sent for paired-end Illumina sequencing (Novogene (UK) Company). In this sequencing method, the resulting raw reads corresponding to each genotype could subsequently be identified using the genotype-specific barcodes incorporated in the forward primers (Supplementary Fig. 1D, E).

### Analysis of alpha-gliadin reads and estimation of relative frequency of CD epitopes

**Fig. 2:**
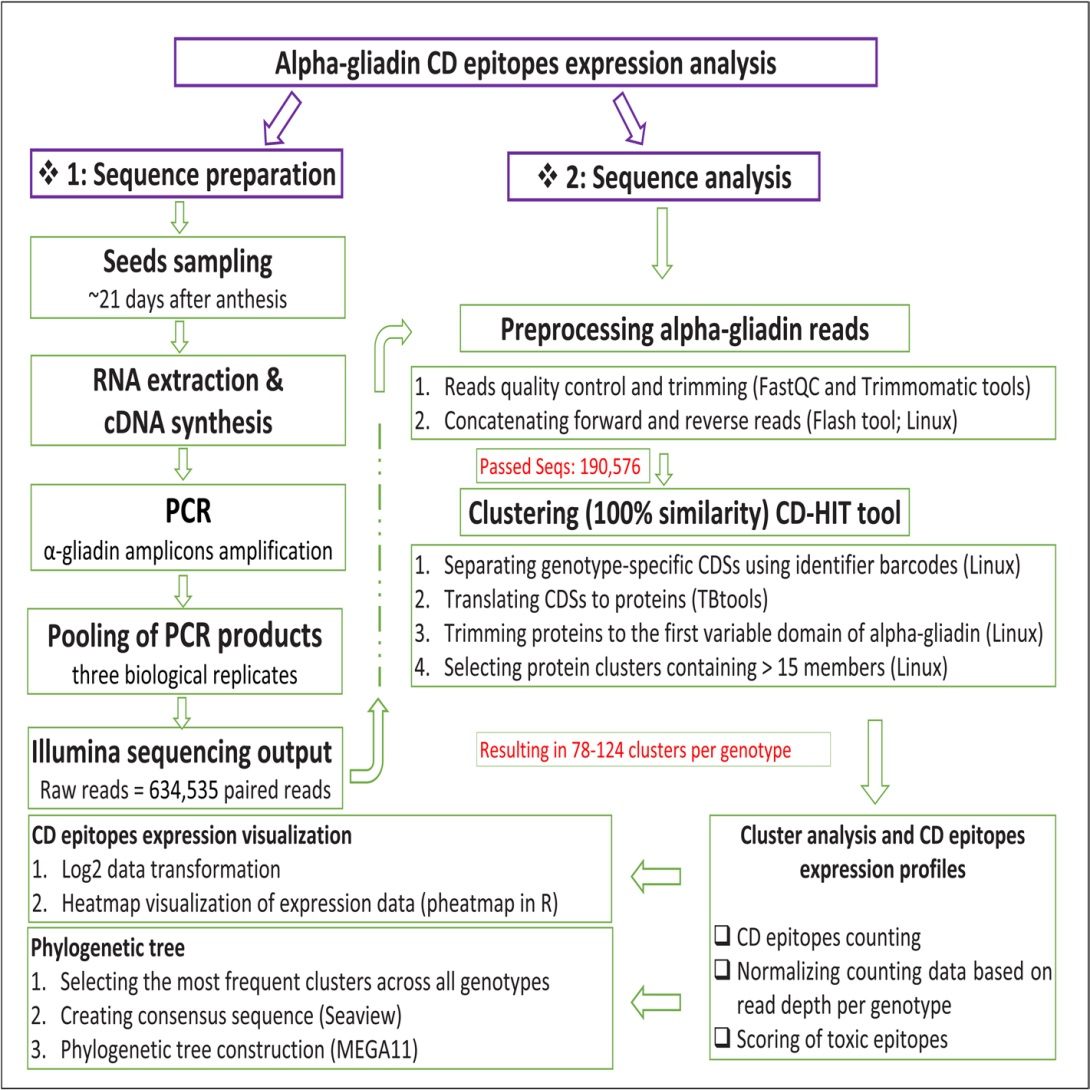
A summary pipeline for preprocessing and analyzing of alpha-gliadin CD epitopes, illustrating the process from raw reads to expression analysis

The overall procedure for preprocessing and analyzing of alpha-gliadin reads is summarized in Fig. 2. A total of 634,535 paired-end reads (with an average length of 250–490 bp per read) were generated from the Illumina sequencing of the pooled alpha-gliadins amplicons (Supplementary Table 1). To assess the overall read quality and remove low-quality bases or unwanted sequences, base quality control and trimming of the alpha-gliadins reads were performed using FastQC and trimmomatic tools, respectively, [20, 21]. The forward and reverse alpha-gliadin fastq reads were then combined/concatenated using FLASH tool [22] in Linux. Out of 634,535 initial paired-end reads, 190,576 were successfully concatenated and retrieved alpha-gliadin amplicons. For each genotype, the corresponding alpha-gliadin amplicons were separated using genotype-specific identification barcodes incorporated into the forward primers (Fig. 1D). The reverse primer contained only the sequence required for Illumina sequencing and was universal across all genotypes (Fig. 1D). To retrieve the alpha-gliadins codding sequences (CDSs), the oligonucleotide sequences of barcodes, signal peptide, and primers were trimmed from the concatenated amplicons using SeqKit toolkit in Linux (Fig. 1B). For each genotype, CDSs were translated into proteins in Tbtools using “Batch Translate CDS to Protein” function [23] and subsequently employed for the downstream frequency analysis of CD epitopes. To achieve this, protein sequences were first clustered using CD-HIT at 100% identity [24] on a Linux platform. Proteins with internal stop codons were identified by an asterisk (*) in their sequences. These proteins were subsequently removed by CD-HIT during clustering. Clusters with more than 15 members were selected as representative sequences for analyzing CD epitope frequency. This threshold was chosen to exclude low-confidence sequences arising from sequencing errors or PCR stochasticity. To assess the relative expression of canonical alpha-gliadin epitopes and their previously identified variants (Supplementary Table 2), we quantified the abundance of each CD epitope within representative clusters and multiplied this by the number of members per cluster. Subsequently, epitopes frequencies were normalized to read depth per genotype (Supplementary Table 3) and reported as relative expression rates. The log2 transformation values of the relative expression data of all CD epitopes were visualized by a heatmap using the pheatmap package [25] in R. To estimate the relative toxicity load of each genotype, we first assigned toxicity scores to different CD epitopes based on reported toxicity rankings from in vitro and clinical experiments [4, 11, 13, 17, 26–30]. Accordingly, highly toxic (HT) and toxic (T) epitopes received a score of 3; moderately toxic (MT) and reduced toxicity (RT) epitopes received a score of 2; and low toxicity (LT) epitopes received a score of 1. The frequency of each epitope was then multiplied by its corresponding score (Supplementary Table 4). Finally, the lines with the highest frequency for all groups of toxic epitopes were considered 100% toxic, and the toxicity load of the remaining lines were estimated relative to this reference line (Supplementary Table 4). Based on the frequency of toxic epitopes, we also generated a new heatmap to display the relative expression levels of each individual toxic epitope across different genotypes (Fig. 4).

### Evolutionary relationships of Aegilops species

We evaluated the evolutionary relationships of the studied *Aegilops* species based on the content and composition of the CD epitopes in the most prevalent representative clusters. For each genotype, the protein sequences of the selected representative clusters (10 clusters) were used to generate a consensus protein using SeaView tool version 4 [31]. The consensus proteins were aligned using ClustalW, and a phylogenetic tree was constructed from the aligned consensus proteins of the studied genotypes using Neighbor-Joining (NJ) method in MEGA11[32] with the following parameters: p-distance, pairwise deletion, and 1000 bootstrap replications.

### Protein banding patterns of alpha-gliadins

To evaluate the banding pattern of alpha-gliadins in the studied *Aegilops* genotypes, gliadin fractions were first extracted from 0.1 g of fresh milled flour using 70% ethanol following the protocol described by K Khan, A Hamada and J Patek [33] with minor modifications [34]. Subsequently, 10 µl of the extracted protein was analyzed using 10% polyacrylamide gel electrophoresis under acidic conditions (Acid-PAGE) [35]. The gliadin banding patterns of Chinese spring wheat [35] were used as an internal control, and the banding patterns of *Aegilops* genotypes were evaluated in comparison to both the control and each other.

## Results

### Alpha-gliadin amplicons analysis

The RNA alpha-gliadin amplicons of ten *Aegilops* genotypes containing D-genome from five distinct species (Table 1) were analyzed for the content, composition, and frequency of alpha-gliadin CD epitopes. For each genotype, the first variable repetitive domain of alpha-gliadin amplicons (Fig. 1B, underlined in blue) was examined for the presence of canonical toxic CD epitopes, including DQ2.5-glia-α1, DQ2.5-glia-α2 and DQ2.5-glia-α3, as well as all previously reported CD epitope variants (Supplementary Table 2). A summary of the overall procedure for analyzing of alpha-gliadin amplicons is presented in Fig. 2.

A total of 634,535 paired-end reads (an average of 62,183 reads per plant, 250–490 bp in size) were generated from the results of multiplex amplicon sequencing of alpha-gliadins. After initial preprocessing of the reads, which included base quality control, trimming, and concatenation of forward and reverse reads, 190,576 alpha-gliadin coding sequences (CDSs, ~ 19,000 sequences per genotype) remained. Of these, 9,234 sequences contained stop codons and excluded from the final analysis. For each plant, alpha-gliadin CDSs were separated by searching for the genotype-specific identifier barcode (8 bp oligonucleotide) (Fig. 1D). The separated CDSs were then translated into proteins in TBtools software and clustered using CD-HIT tool with a threshold of 100% similarity. A total of 75,525 clusters were produced from 181,342 CDSs across all genotypes. Clusters with greater than 15 members were selected for CD epitopes expression analysis. Consequently, 1,031 clusters remained, with an average of 78 to 123 clusters per genotype (Supplementary Table 1; Fig. 2).

### Distribution and expression analysis of alpha-gliadin CD epitopes

To analyze the expression patterns of alpha-gliadin CD epitopes across various *Aegilops* species, first, a list of 147 previously identified CD epitopes, including the canonical forms and all those variants with one or two mismatches along with their toxicity information were retrieved from the previous literatures [4, 11, 13, 17, 26–30]. These epitopes were then searched within the selected protein clusters corresponding to each genotype to assess their presence and determine their frequencies across different genotypes. A total of 40 CD epitopes were identified in the studied genotypes (Table 2; Supplementary Table 3). Among these, six epitopes were the canonical epitopes of DQ2.5-glia-α1a or DQ2.5-glia-α1b, and their corresponding variants. Nine epitopes include DQ2.5-glia-α2 and its variants, among these, three variants contained one mismatch, while the remaining variants exhibited two mismatches. Additionally, nine epitopes were DQ2.5-glia-α3 and its variants, among these, four variants contained one mismatch, while the remaining variants exhibited two mismatches. Thirteen epitopes were p31-43 and its variants. Furthermore, three additional epitopes were identified in certain genotypes, including two variants from DQ2.5-ave-1b (with the canonical peptide of PYPEQEQPF specifically reported in oat), and a variant from DQ2.5-glia-g1 (with the canonical peptide of PQQSFPEQQ).

**Table 2:**
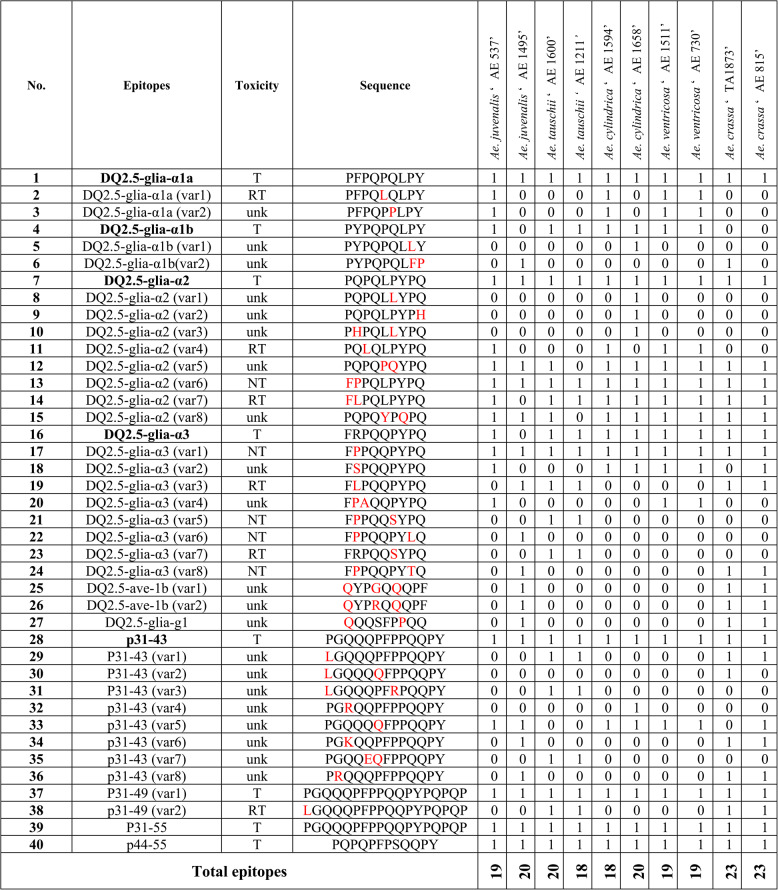
A list of canonical alpha-gliadin CD epitopes (indicated in bold), their related variants and toxicity information from a panel of ten *Aegilops* genotypes

Numbers 1 and 0 in the columns beneath the genotypes indicate whether the respective epitopes are present (1) or absent (0) in the alpha-gliadin CDSs of the corresponding species. The amino acid residue of the variants with one, two, or three mismatches are highlighted in red. The toxicity levels of the epitopes are indicated using specific symbols: HT/T for highly toxic or toxic epitopes, MT/RT for moderately toxic or reduced-toxicity epitopes, LT for low-toxicity epitopes, NT for non-toxic epitopes, and unk for epitopes with unknown toxicity

**Fig. 3:**
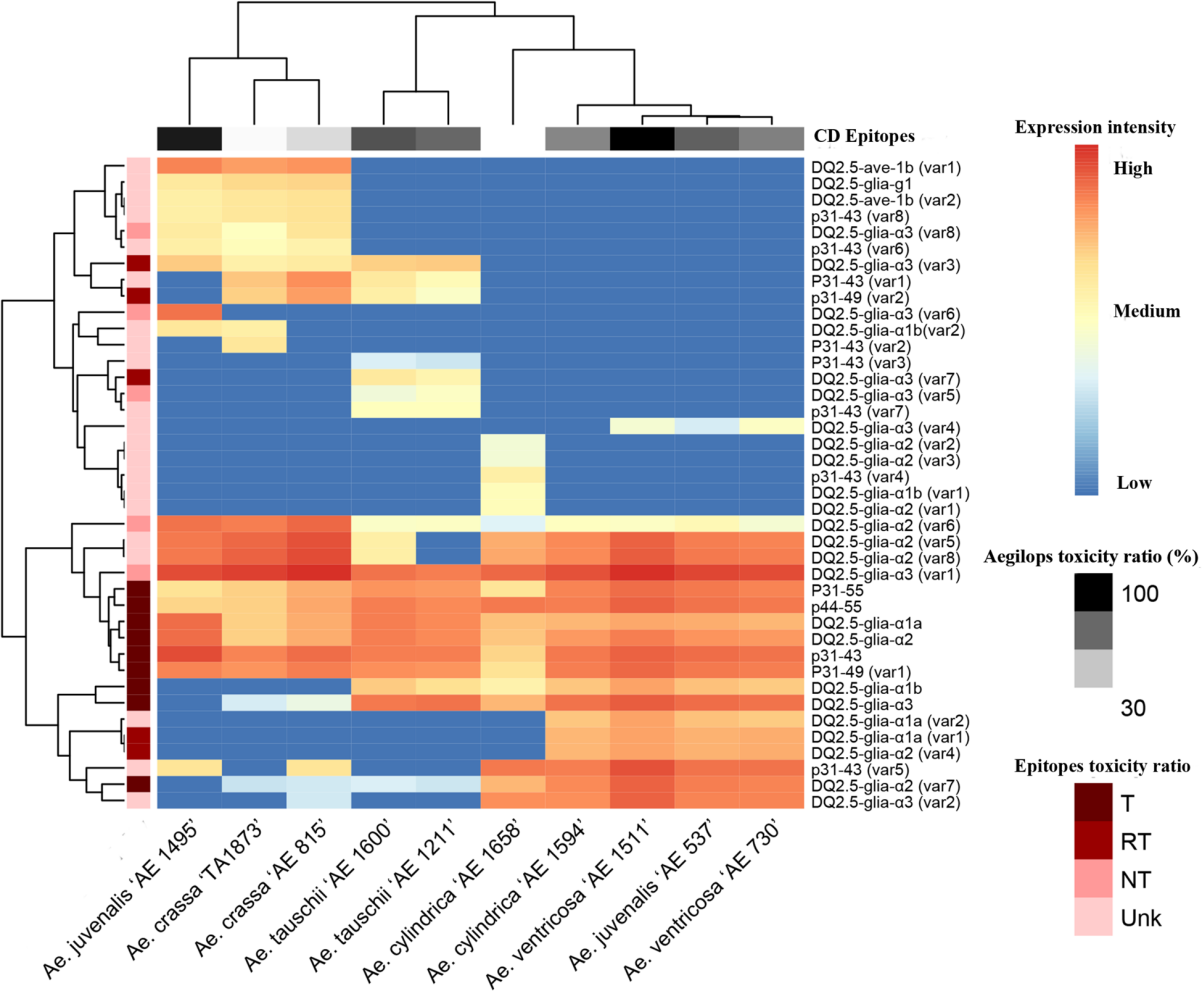
Expression profiles of the canonical alpha-gliadin CD epitopes and their variants in the studied *Aegilops* genotypes (Table 1). The epitope toxicity is shown in brown color ranges (T for toxic, RT for reduced toxicity, NT for non-toxic, and Unk for unknown epitopes). Species were clustered based on the collective frequency of the T and RT epitopes in black range color; black (highly toxic) to white (less toxic)

The distribution patterns and expression profiles of CD epitopes varied considerably among different *Aegilops* species. All the canonical epitopes including the major immunogenic CD epitopes (DQ2.5-Glia-α1a (PFPQPQLPY), DQ2.5-Glia-α1b (PYPQPQLPY), DQ2.5-Glia-α2 (PQPQLPYPQ), and DQ2.5-Glia-α3 (FRPQQPYPQ)) were expressed across all *Aegilops* genotypes though with different frequencies. Among these, P31-43 showed the highest frequency across genotypes, with an average of 3,257 epitopes. The relative frequencies of the remaining canonical epitopes followed the order: DQ2.5-glia-α3 > DQ2.5-glia-α2 > DQ2.5-glia-α1a > DQ2.5-glia-α1b. The canonical epitopes of DQ2.5-glia-α1b and DQ2.5-glia-α3 were not detected in certain species (Table 2; Supplementary Table 3). For example, DQ2.5-glia-α1b was absent across all accessions of *Ae. crassa* and in one accession of *Ae. juvenalis* ‘AE 1495’. Moreover, DQ2.5-glia-α3 exhibited the lowest frequencies among all *Ae. crassa* accessions (e.g., a frequency of 22 in *Ae. crassa* ‘TA1873’ and 45 in *Ae. crassa* ‘AE 815, compared with an overall average frequency of 2020 across all *Aegilops* accessions) and was also absent in the same *Ae. juvenalis* ‘AE 1495’ accession (Table 2; Supplementary Table 3; Fig. 3). Among the different *Aegilops* species, P31-43 exhibited the highest frequency in *Ae. juvenalis* ‘AE 1495’ and the lowest in *Ae. cylindrica* ‘AE 1658’. Both DQ2.5-glia-α1b and DQ2.5-glia-α3 were most frequent in *Ae. ventricosa* ‘AE 1511’ and least frequent in *Ae. crassa* accessions and *Ae. juvenalis* ‘AE 1495’. Similarly, DQ2.5-glia-α1a and DQ2.5-glia-α2 revealed their highest frequencies in *Ae. juvenalis* ‘AE 1495’ and their lowest in *Ae. crassa* ‘TA1873’ (Table 2; Supplementary Table 3; Fig. 3).

Among the 40 identified CD epitopes, 18 displayed elevated expression profiles across the studied genotypes. These comprised DQ2.5-glia-α1a and its two variants [DQ2.5-glia-α1a (var1) and DQ2.5-glia-α1a (var2)]; the canonical DQ2.5-glia-α1b; DQ2.5-glia-α2 and its five variants [DQ2.5-glia-α2 (var4), (var5), (var6), (var7), and (var8)]; DQ2.5-glia-α3 and its two variants [DQ2.5-glia-α3 (var1) and (var2)]; as well as p31-43 and its four corresponding variants [p31-43 (var1), p31-43 (var5), p31-55, and p44-55]. Of these 18 epitopes, 12 were consistently expressed across all genotypes, while the remaining six [DQ2.5-glia-α1a (var1), DQ2.5-glia-α1a (var2), DQ2.5-glia-α2 (var4), DQ2.5-glia-α2 (var7), DQ2.5-glia-α3 (var2), and p31-43 (var5)] exhibited genotype-specific expression, being largely detected in *Ae. cylindrica* ‘AE 1658’, *Ae. cylindrica* ‘AE 1594’, *Ae. ventricosa* ‘AE 1511’, *Ae. juvenalis* ‘AE 537’, and *Ae. ventricosa* ‘AE 730’ (Supplementary Table 3; Fig. 3). The remaining 22 epitopes were detected only in specific genotypes and at comparatively low frequencies.

**Fig. 4:**
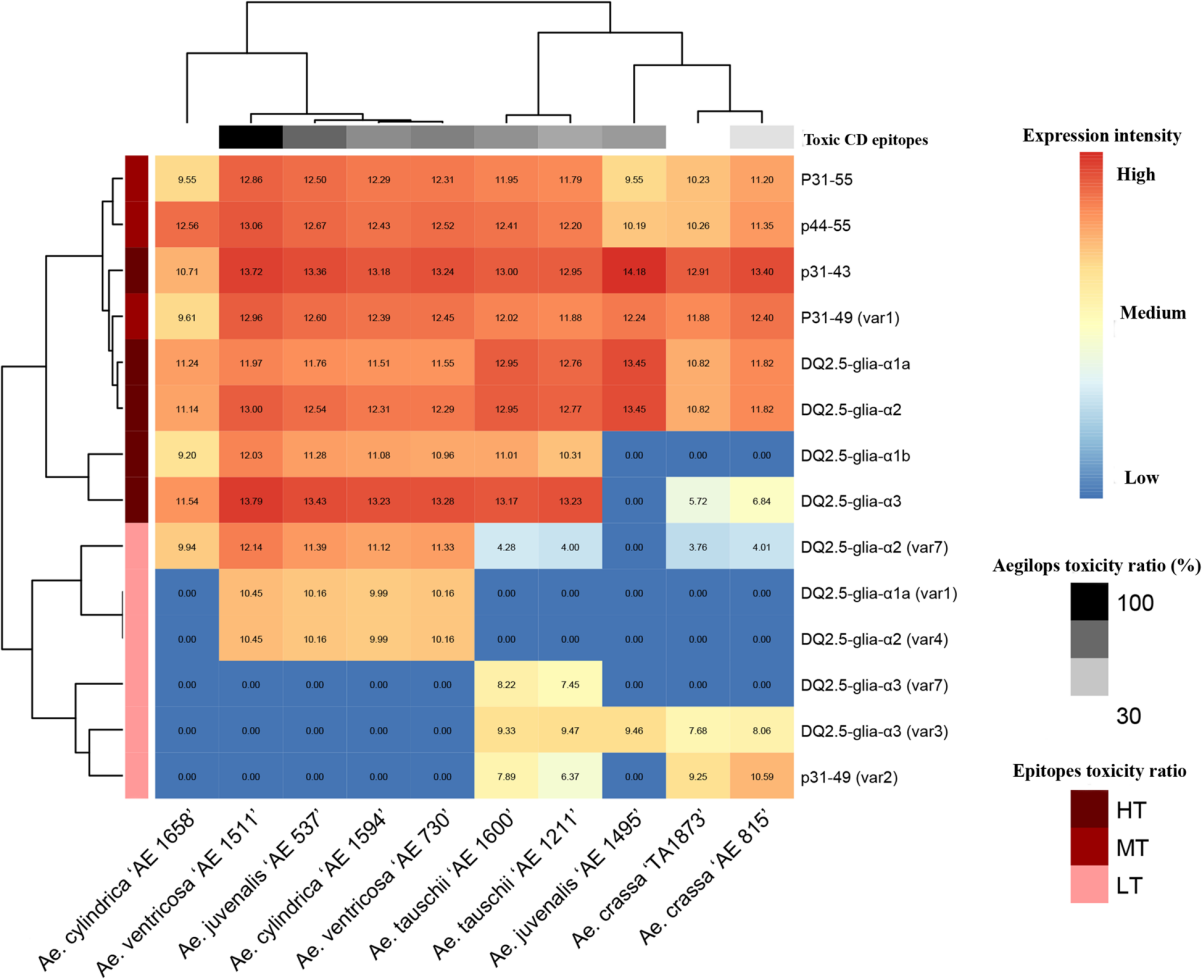
The log 2 transformed expression profiles of toxic CD epitopes in the studied *Aegilops* genotypes (Table 1). The epitope toxicity is shown in brown color ranges (HT for highly toxicity, MT for moderately toxicity, and LT for low toxicity). Species were clustered based on the overall frequency of toxic epitopes in black range color; black (highly toxic) to white (less toxic)

Based on the previously reported toxicity information of various CD epitopes (their relative toxicity rankings for inducing CD [4, 11, 13, 17, 26–30]), and the relative frequency of toxic epitopes in the current study (Supplementary Table 4), the immunogenicity levels of different *Aegilops* species were estimated. Accordingly, the epitopes were first classified into three groups. Group I comprised the major CD epitopes, including the canonical DQ2.5-glia-α1a, DQ2.5-glia-α1b, and DQ2.5-glia-α2 epitopes which were designated as highly toxic (HT/T). Group II included epitopes with reduced toxicity, namely DQ2.5-glia-α3, p31-49 (var1), p31-55, and p44-55, classified as moderately toxic (MT/RT). Group III encompassed epitopes with comparatively low toxicity, including DQ2.5-glia-α1a (var1), DQ2.5-glia-α2 (var4), DQ2.5-glia-α2 (var7), DQ2.5-glia-α3 (var3), DQ2.5-glia-α3 (var7), and p31-49 (var2), which were categorized as low toxic (LT). To evaluate the relative expression profiles of CD epitopes across genotypes, the normalized expression values of each epitope group (HT, MT, and LT) were first weighted according to their relative toxicity rank for inducing CD by applying scores of 3, 2, and 1, respectively. The weighted values were then summed to estimate the overall toxicity level for each genotype (Fig. 4; Supplementary Table 4). The toxicity level of each *Aegilops* species was expressed as a percentage relative to that of the species with the highest toxicity score, which was set at 100%. Log2-transformed expression data of CD epitopes (Fig. 4) revealed distinct toxicity patterns among *Aegilops* species. *Ae. ventricosa* ‘AE 1511’ contained the highest expression frequency for almost all groups of CD epitopes and considered the first toxic species (100% toxic). *Ae. juvenalis* ‘AE 537’ contained the highest expression frequency for p31-43 and DQ2.5-glia-α3, and identified as the second toxic species (75%). Species of *Ae. tauschii* ‘AE 1211’, *Ae. cylindrica* ‘AE 1594’, *Ae. tauschii* ‘AE 1600’, *Ae. ventricosa* ‘AE 730’, and *Ae. juvenalis* ‘AE 1495’ contained the highest expression frequency for two to four of DQ2.5-glia-α1a, DQ2.5-glia-α2, DQ2.5-glia-α3, and p31-43 epitopes, had the approximately toxicity percentage of 60–69%. The species with the lowest toxicity percentage included *Ae. crassa* ‘TA1873’ (26%), *Ae. cylindrica* ‘AE 1658’ (25%), and *Ae. crassa* ‘AE 815’ (43%), where DQ2.5-glia-α1a and DQ2.5-glia-α2 were the most abundant epitopes (Fig. 4; Supplementary Table 3).

In addition to evaluating the expression profiles of each of these toxic CD epitopes individually, we also searched for the presence of the intact 33-mer region (LQLQPFPQPQLPYPQPQLPYPQPQLPYPQPQP [AFX69628.1]; which associated with higher CD toxicity in common wheat) in the alpha-gliadin CDSs across various *Aegilops* species. This intact form of the 33-mer region was not detected in any of the studied genotypes.

**Fig. 5:**
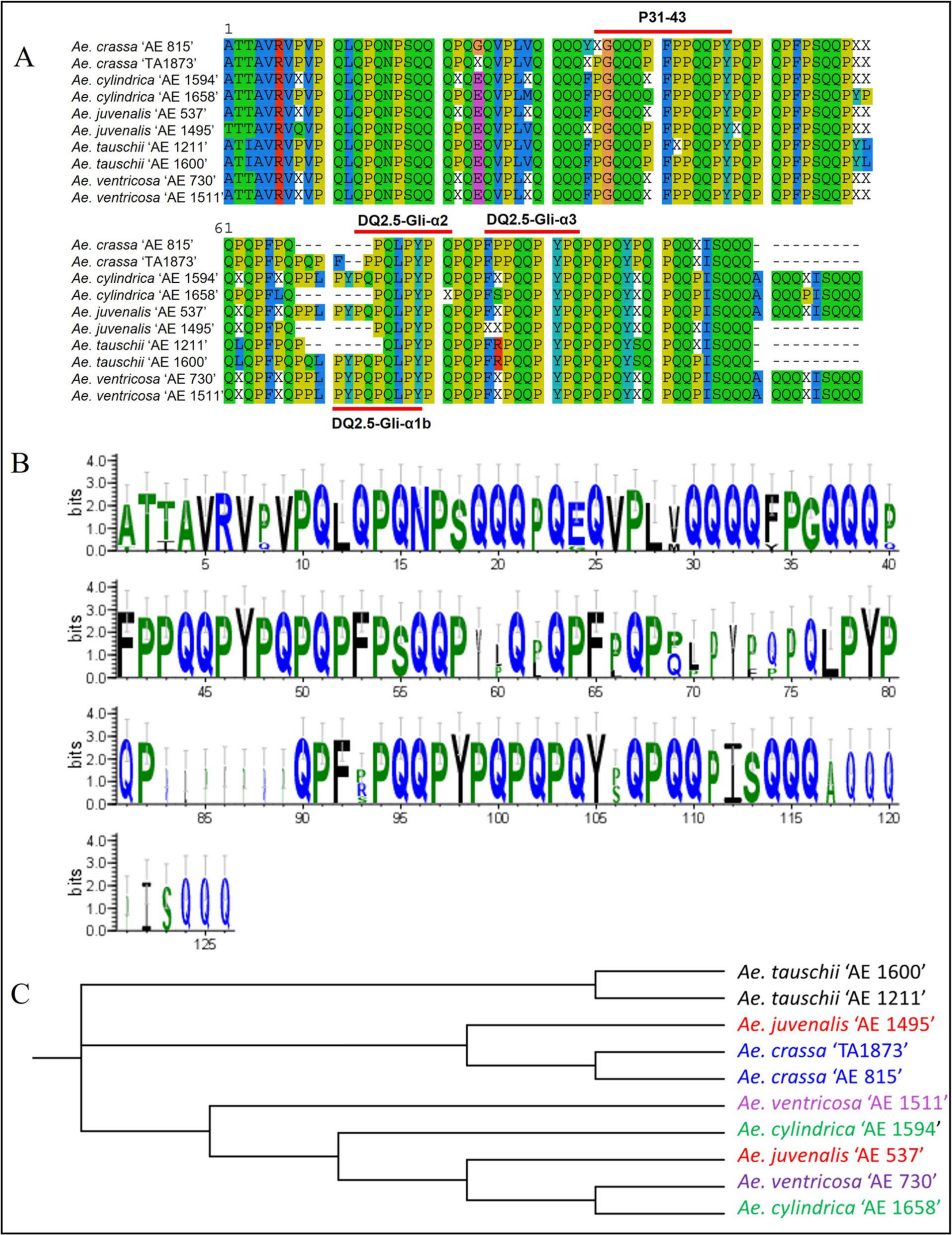
Analysis of alpha-gliadin consensus sequences in different *Aegilops* species. **A** Alignment pattern of consensus sequences of the ten prevalent alpha-gliadin amplicons in *Aegilops* genotypes. The alignment status shows that P31-43 and DQ2.5-Glia-α3 peptides are highly conserved across all *Aegilops* species, while DQ2.5-Glia-α1b and DQ2.5-Glia-α2 are conserved only in some species. **B** A sequence logo of aligned prevalent alpha-gliadin proteins. **C** Evolutionary relationships of different *Aegilops* species according to the common alpha-gliadin proteins in each genotype

Based on the alignment and logo patterns of consensus sequences of the prevalent alpha-gliadin amplicons, the conservation of canonical CD epitopes was evaluated across all *Aegilops* species. P31-43 and DQ2.5-glia-α3 showed a relatively conserved amino acid pattern across all *Aegilops* species, while DQ2.5-glia-α1b and DQ2.5-glia-α2 epitopes were only conserved in some species (Fig. 5A, B). The evolutionary tree constructed using these consensus alpha-gliadin amplicon sequences grouped *Aegilops* genotypes in three distinct clades, with the same *Aegilops* species in the same clade or in relatively close branches within the neighboring clades (Fig. 5C).

### Acid-PAGE banding patterns of gliadins

**Fig. 6:**
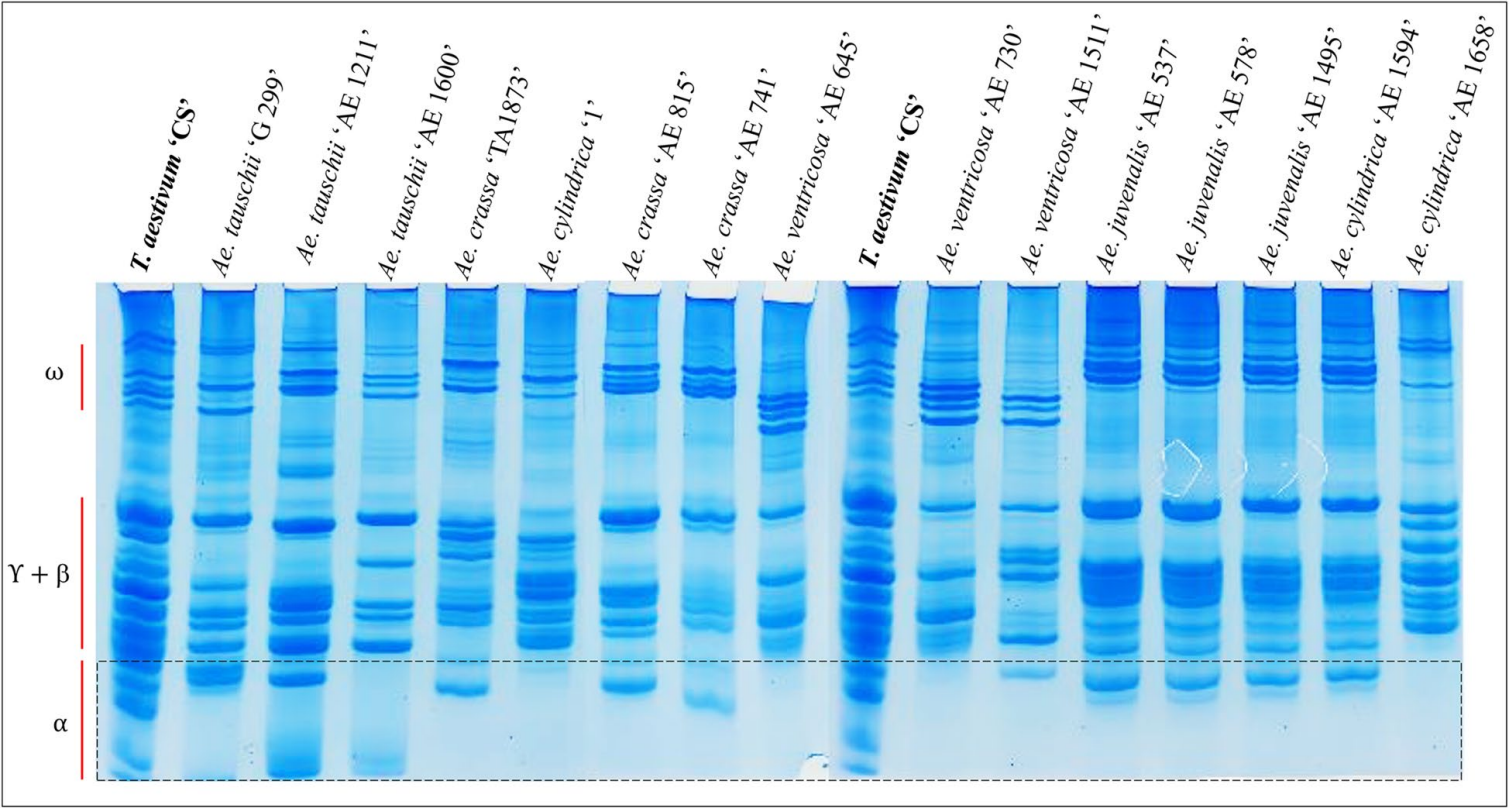
Acid-PAGE gliadin banding patterns in different *Aegilops* genotypes. The lane for *T. aestivum* ‘CS’ shows gliadin banding pattern in Chinese spring wheat (Control line). The positions of the different fractions of gliadins (ω, γ/β-, and α-gliadins) is indicated beside the first lane with red lines. A dashed rectangle indicates the position of alpha-gliadin bands across various studied *Aegilops* genotypes

Though the banding patterns of ω-, γ + β-gliadins were not our focus in this research, they exhibited high differences among different *Aegilops* genotypes (Fig. 6). Regarding alpha-gliadins, their patterns not only varied among the different *Aegilops* species, but also between different accessions of the same species. In general, no alpha-gliadin bands were detected in *Ae. cylindrica* ‘AE 1658’ and *Ae. ventricosa* ‘AE 730’, while other genotypes had one or two alpha-gliadin bands (Fig. 6).

## Discussion

Among gluten proteins, alpha-gliadins exhibit the strongest CD immunogenicity since they posses the canonical T-cell stimulatory epitopes (DQ2.5-glia-α1, DQ2.5-glia-α2, and DQ2.5-glia-α3) responsible for eliciting the immunogenicity in genetically susceptible individuals [36]. Although these immunogenic epitopes are found across different wheat subgenomes, they primarily originate from the D subgenome. Thus, given the importance of *Aegilops* D subgenome in wheat breeding programs and its role in enhancing the genetic diversity of wheat gluten proteins [37–39], we aimed to analyze the transcriptome of alpha-gliadin amplicons from ten *Aegilops* genotypes to evaluate their content, composition, and frequency of alpha-gliadin CD epitopes. These genotypes belong to five distinct species, all of which contained the D subgenome in their pedigree (Table 1).

After preprocessing the alpha-gliadin paired-end reads, approximately one-third of the reads (181,342) reconstructed the alpha-gliadin coding sequences (CDSs). This high reduction in the reconstructed reads was partly due to the applying quality control filter on reads, the concatenation of forward and reverse reads (which excluded the incomplete or partial reads), and the filtering of CDSs containing stop codons. Despite this reduction, the remaining number of CDSs are high enough to assess the relative distribution and frequency of celiac disease (CD) epitopes across different studied *Aegilops* genotypes. Therefore, the subsequent analyses were conducted using these CDSs.

Out of the 147 previously identified CD epitopes, 40 were detected in the alpha-gliadin amplicons of the *Aegilops* genotypes analyzed in this study. On average, 18 to 23 epitopes were identified in each line. Compared with the alpha-gliadin CD epitopes reported in a panel of eight synthetic wheat lines [18], the number of CD epitopes in our *Aegilops* genotypes is approximately half. Overall, aside from the canonical epitopes, the type and variant of CD epitopes present in the two panels differed substantially. Further analysis of alpha-gliadin epitopes revealed that none of the *Aegilops* genotypes contained the intact 33-mer highly toxic peptide. However, based on our previous study [18], a synthetic wheat line, developed from the same accession of *Ae. crassa* ‘1873’ studied here, contained a very low quantity of the intact 33-mer peptide. This discrepancy in the current results may stem from inefficient PCR amplification of low-abundance amplicons or the stringent filtering applied during alpha-gliadin clustering, which excludes low-quantity amplicons to ensure high-confidence results. Furthermore, it is well-established that significant genetic variation can occur between different sub-accessions of plant material, potentially influencing genome composition [40, 41].

Despite the strong differences in the types and distribution patterns of identified CD epitopes among the *Aegilops* genotypes, all canonical and toxic CD epitopes were detected in every line examined. This suggests that none of these species are entirely safe for patients susceptible to CD. However, since a lower toxicity level in a specific line correlates with a reduced frequency of toxic CD epitopes in its gluten content [26, 42], it is possible to evaluate different *Aegilops* genotypes in terms of their overall toxicity load. Therefore, to assess this load, we measured not only the frequency of the canonical epitopes but also the frequency of other previously identified toxic variants. To increase the accuracy of this evaluation, we assigned scores ranging from 3 (highest toxicity) to 1 (lowest toxicity) to the frequency of these toxic epitopes, based on their previously reported toxicity rankings from in vitro and clinical experiments [4, 11, 13, 17, 26–30]. This scoring system enables a more precise estimation of the relative toxicity load of the lines. However, our results were largely consistent even without this scoring system, highlighting considerable differences among *Aegilops* genotypes in their incorporation of toxic epitopes into their genomes.

A deeper analysis of the data demonstrated that different accessions within the same *Aegilops* species sometimes exhibited varying expression patterns for toxic CD epitopes, leading to their classification under different toxicity levels. For instance, *Ae. ventricosa* ‘AE 1511’ displayed the highest expression profile for all toxic CD epitopes, identifying it as the most toxic genotype (100% toxicity). In contrast, the other accession of this species, *Ae. ventricosa* ‘AE 730’, showed a lower expression pattern for these epitopes, classifying it as a genotype with a medium toxicity load (60–69%). Similarly, different accessions of *Ae. cylindrica* were also categorized into distinct toxicity levels: ‘AE 1594’ exhibited medium toxicity, while ‘AE 1658’ showed low toxicity (25–43%). Notably, only accessions from *Ae. tauschii* and *Ae. crassa* genotypes displayed similar patterns of toxic epitope integration in their genomes, and were consequently classified under the medium and low toxicity group, respectively.

The Acid-PAGE analysis of gliadin revealed that genotypes with lower toxicity loads either lacked alpha-gliadin bands (e.g., *Ae. cylindrica* ‘AE 1658’) or contained only a single band (e.g., both accessions of *Ae. crassa*). However, the number of alpha-gliadin bands did not consistently correlate with the toxicity load of the studied genotypes. For instance, the species with a higher toxicity load, such as *Ae. ventricosa* ‘AE 1511’, also exhibited just one alpha-gliadin band. In some cases, species with medium toxicity loads displayed two alpha-gliadin bands (e.g., *Ae. tauschii* ‘AE 1211’) (Fig. 6).

Taken together, we conclude that the distribution and frequency of CD epitopes differ considerably not only in distinct *Aegilops* species but also among accessions within the same species. In the panel of *Aegilops* genotypes we studied, none contained the full 33-mer highly toxic region. Similarly, previous studies reported that in most *Aegilops* genotypes, the α-gliadins lacked an intact 33-mer peptide [13, 18], and therefore suggested their potential use in developing wheat varieties with fewer or no celiac disease epitopes through the creation of synthetic hexaploid wheats. By comparing the relative frequencies of CD epitopes among different *Aegilops* species, we found that both *Ae. crassa* accessions and *Ae. cylindrica* accession ‘AE 1658’, despite lacking the highly toxic intact 33-mer peptide, contained the lowest number of toxic CD epitopes. Based on this information, the utilization of the D genome from these accessions in wheat breeding programs may be a promising strategy to reduce CD toxicity in the developed synthetic wheats. Consistent with our findings, in a panel of synthetic wheat lines [18], where the lines were derived from *Ae. crassa* D subgenome, their toxicity load of CD epitopes was much lower than that of lines derived from other *Aegilops* species. However, developing synthetic wheats from *Aegilops cylindrica* remains challenging, likely due to high rates of or complete hybrid sterility [43, 44]. Furthermore, several other key factors beyond CD toxicity must also be addressed when creating synthetic wheat from these accessions, such as acceptable yield or retaining bakery functionality while reducing CD allergenicity.

## Conclusion

In the present study, ten different genotypes of the *Aegilops* genus representing five distinct species including *Ae. tauschii*, *Ae. crassa*, *Ae. juvenalis*, *Ae. cylindrica*, and *Ae. ventricosa* were assessed for the content, composition and relative frequency patterns of CD epitopes in the most toxic region of alpha-gliadin genes. The different species exhibited considerable variation in the frequency and type of CD epitopes. *Ae. tauschii* genotypes, which have played a major role in the evolution of common wheat, were categorized within the medium toxicity load group. Both accessions of *Ae. crassa* and one accession of *Ae. cylindrica* (‘AE 1658’) contained lower amounts of toxic CD epitopes comparing to other *Aegilops*, suggesting they may serve as potential candidate species for developing synthetic wheats with reduced gluten allergenicity for individuals susceptible to celiac disease.

## Supplementary Information

Supplementary Material 1. Supplementary Table 1. Overall information of the results of alpha-gliadins amplicon sequencing.

Supplementary Material 2. Supplementary Table 2. Canonical alpha-gliadin CD epitopes, all previously identified variants, and their corresponding amino acid sequences.

Supplementary Material 3. Supplementary Table 3. The frequency of the canonical and all variants of alpha-gliadin CD epitopes across different *Aegilops* genotypes.

Supplementary Material 4. Supplementary Table 4. Estimation of the relative toxicity of different *Aegilops* genotypes based on the presence and frequency of toxic alpha-gliadin CD epitopes in their alpha-gliadin amplicons.

Supplementary Material 5. Supplementary Fig. 1. Agarose gel electrophoresis of RNA and PCR products from alpha-gliadin amplicons in different *Aegilops* genotypes. A Agarose gel electrophoresis of RNA samples extracted from *Aegilops* seeds at 21 days post-anthesis. B Electrophoresis bands of alpha-gliadin PCR products amplified from cDNA using alpha-gliadin primers (E, blue sequences; First PCR). C Electrophoresis bands of alpha-gliadin PCR products amplified using the first PCR product as template and fusion primers (E, for fusion primers see longer sequences). D *Aegilops* genotypes studied in this research. E Alpha-gliadin and fusion primers. The original alpha-gliadin primers are shown in blue. Each forward fusion primer contains the adapter sequence for Illumina preparation library (in black), 8 bp barcode sequence specific to each *Aegilops* genotype (in red), and the original alpha-gliadin forward primer (in blue). The reverse fusion primer is universal and contains only the Illumina adapter sequence and the original alpha-gliadin reverse primer.

## Data Availability

All relevant data are within the manuscript and its Supporting Information files. The alpha-gliadin amplicon sequencing reads are available in the NCBI Sequence Read Archive (SRA) under accession number PRJNA1419916. https://www.ncbi.nlm.nih.gov/bioproject/PRJNA1419916.
