# Supplementary figures and images for: Evaluation of alpha-gliadin celiac disease epitopes in some Aegilops species containing the D genome
Source: BMC Plant Biol. 2026 May 20;26:969. doi: 10.1186/s12870-026-08966-0 (PMC13235123; doi:10.1186/s12870-026-08966-0)

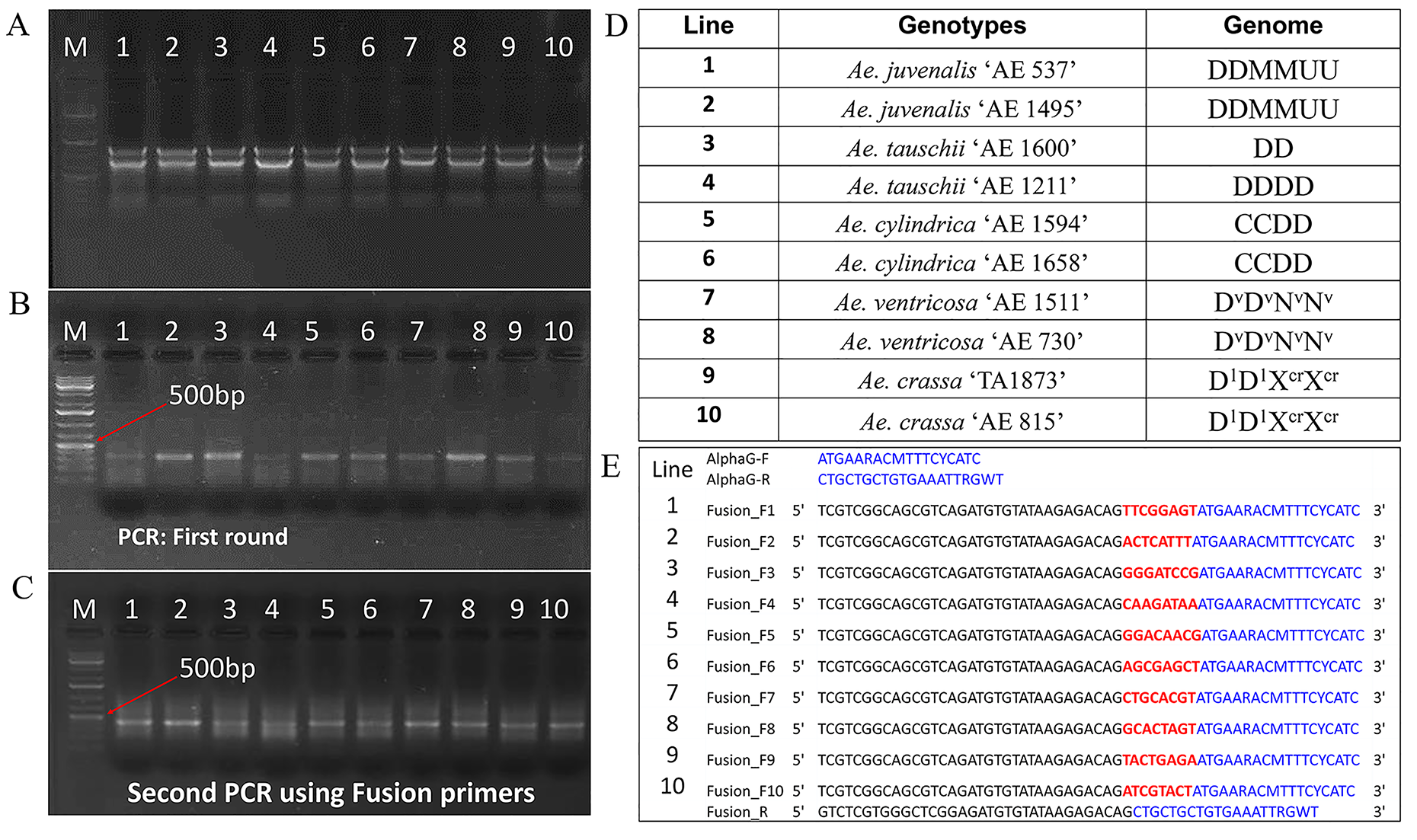

Supplement: Supplementary file 1 — Supplementary Material 1. Supplementary Table 1. Overall information of the results of alpha-gliadins amplicon sequencing. [file 12870_2026_8966_MOESM1_ESM.tif]
